# Psychometric properties and confirmatory factor analysis of the Jefferson Scale of Physician Empathy

**DOI:** 10.1186/1472-6920-11-54

**Published:** 2011-08-02

**Authors:** Sina Tavakol, Reg Dennick, Mohsen Tavakol

**Affiliations:** 1 School of Biomedical Sciences, Queen's Medical Centre, The University of Nottingham, Nottingham, NG7 2UH, UK; 2 Medical Education Unit, Queen's Medical Centre, The University of Nottingham, Nottingham, NG7 2UH, UK

**Keywords:** Empathy, JSPE, confirmatory factor analysis, structure validation

## Abstract

**Background:**

Empathy towards patients is considered to be associated with improved health outcomes. Many scales have been developed to measure empathy in health care professionals and students. The Jefferson Scale of Physician Empathy (JSPE) has been widely used. This study was designed to examine the psychometric properties and the theoretical structure of the JSPE.

**Methods:**

A total of 853 medical students responded to the JSPE questionnaire. A hypothetical model was evaluated by structural equation modelling to determine the adequacy of goodness-of-fit to sample data.

**Results:**

The model showed excellent goodness-of-fit. Further analysis showed that the hypothesised three-factor model of the JSPE structure fits well across the gender differences of medical students.

**Conclusions:**

The results supported scale multi-dimensionality. The 20 item JSPE provides a valid and reliable scale to measure empathy among not only undergraduate and graduate medical education programmes, but also practising doctors. The limitations of the study are discussed and some recommendations are made for future practice.

## Background

Empathy, the ability to effectively grasp patients' emotional needs in the context of patient care, is considered to be associated with improved health outcomes [1-4]. Over the past decade, medical educators and professional bodies have increasingly expressed concerns for the humanistic values and the enhancement of interpersonal skills among medical students [3-7]. The Association of American Medical Colleges recommended that empathy should be integrated and assessed in graduate medical education [8]. Crucial to the measurement of empathy is the availability of an instrument that can validly measure the empathy of medical students.

Although twenty instruments have been employed to measure the empathy levels of healthcare professionals [9], the Jefferson Scale of Physician Empathy (JSPE) has been specifically constructed in the context of the doctor-patient relationship and patient care [10]. Over the past ten years, the JSPE has been used in several settings to measure empathy among not only undergraduate and graduate students, but also practicing doctors [1,11-25]. The JSPE enables medical educators to "evaluate the effectiveness of educational interventions aimed at promoting empathy". It can also be used to examine the variation and correlation of empathy in different years of medical education and between genders [13].

The JSPE is undoubtedly the most widely used measure of empathy in the context of patient care and has been translated into 25 languages [26]. Nevertheless, its construct validity for use by medical educators to assess medical students has not been firmly established.

The seminal work of empathy researchers showed that empathy was a multidimensional model comprising three related constructs: (a) perspective taking; (b) compassionate care; and (c) ability to stand in patient's shoes [17,18,27]. These dimensions (factors) have been empirically produced using explanatory factor analysis (EFA). A limitation, noted with respect to previous validity research, is that the JSPE has only been examined using an EFA approach based on principle component analysis. It has been well documented that there are several deficiencies associated with the EFA approach to determine the validity of latent constructs/concepts of a scale (factor validity) [28].Thus, the primary purpose of this study was to test the psychometric properties and the theoretical structure of the JSPE among UK medical students via both EFA and confirmatory factor analysis (CFA).

### Factor analysis

Factor analysis is the most powerful statistical procedure for scrutinising relations between observed and latent variables. To this end, researchers investigate the correlated variation among a set of observed variables in order to glean information from their underlying latent variables (factors/constructs). There are two types of factor analysis: EFA and CFA. A brief explanation of EFA and CFA may facilitate understanding of utilisation of CFA in this study.

When the researcher is not aware of the connections between the observed and latent variables, the EFA approach describes how and to what extent the observed variables are related to their latent constructs. The number of factors that have been generated from a set of items in a study using EFA is termed the factor structure or model. For example, if a study has generated 3 factors, the model is termed the three-factor model. This is in contrast to CFA, which is used when researchers have prior knowledge of latent (underlying) variables and seek to confirm factors that they have found using EFA. The EFA approach is a data-driven approach in which a model or theory is created whereas CFA is a theory/model driven approach where a model or theory is tested. CFA thus departs from EFA in that researchers must first identify a factor model before analysing data.

## Methods

### Participants and procedures

We conducted this study between March 2009 and February 2010. The study cohort represents 68.24% (n = 853) of the total medical students at the University of Nottingham, UK, encompassing both males (n = 351) and females (n = 470). Some students (n = 32) did not indicate their gender in the study. No financial reward was provided for students wishing to participate in the study.

### Measure of study

The JSPE is a self-administrated 20-item scale designed to measure empathy in the context of patient care and doctor-patient relationship [1,29]. The questionnaire takes 5 minutes to complete. Students rate their level of empathy for each item on the JSPE from 1 (strongly disagree) to 7 (strongly agree), with higher scores indicating higher levels of empathy.

### Procedure

Following ethical approval granted by University of Nottingham Medical School Ethics Committee, the JSPE was recast in the form of a web application via Adobe Flash. A confidential hyperlink to the questionnaire was then placed on the Networked Learning Environment of the University of Nottingham. Access to questionnaire was therefore only granted to medical students at this university. The items were not mandatory and students had the option of abstaining from each question. The application stored the information collected from the study within a secure database. This data was subsequently downloaded directly into SPSS 17 for further analysis.

### Statistical analysis

Responses from medical students to the scale were coded and entered into SPSS 17. Demographic missing data were coded as missing and excluded from relevant analysis. We replaced items of missing data with the mean. However, those students who did not provide a response to four or more items were not included in subsequent analysis. Based on this, 54 (5.30%) students were excluded from the study. Descriptive analyses were performed on all items.

As we do not have an idea of the underlying components of the JSPE in the UK, we performed Principle Component Analysis (PCA) to explore the links between the observed variables (items) and the latent variables (factors) and to identify the factor structure. The nature of PCA is exploratory rather than confirmatory [30]. We retained only factors with eigenvalues greater than 1.25 [31]. Factor coefficients of 0.40 or greater were required for the interpretation of the factor structure [32]. A Cronbach's alpha of > 0.70 is considered to be an acceptable reliability coefficient for determining the internal consistency of the scale [33]. Corrected-item total correlation (the degree to which each item correlates with the total score) was performed to identify items that are problematic and need to be revised or discarded. Pearson's correlation coefficients were calculated to investigate the inter-relationships between the JSPE dimensions. Known group validity (the ability of a scale to distinguish participants of one group from another group based on their responses to the scale) was assessed by comparing gender groups using t-tests.

Structural Equation Modelling (SEM) was performed to evaluate relationships between structural paths and latent variables (factors) using AMOS 17. SEM is a confirmatory technique in contrast to PCA [30]. Data analysis was carried out in two steps. First, to facilitate interpretation, responses for negative items were reversed. Second, the multidimensionality of the structural model proposed by this study was tested by total sample and gender for its fit with the observed covariance structure of the measured items.

### Assessing the degree of model fit

We assessed the parameters of the model using AMOS 17. Five goodness-of-fit indices were calculated in order to assess global fit of the model by total sample and gender. These indices include: χ^2 ^and its subsequent ratio with degrees of freedom (χ^2^/df); goodness-of-fit index (GFI); adjusted GFI; comparative fit index (CFI) and root mean square error of approximation (RMSEA). The chi-square statistic is calculated to assess the fit between the hypothesised statistical model and the set of observed variables (items). A statistically significant chi-square test suggests that the model has a lack fit to data. In addition to the chi-square statistic, a range-of-fit statistics (such as GFI) was calculated to describe how well the model fits the set of observed data. The GFI shows the degree of variance and covariance together explained by the model. The value of GFI ranges between 0 and 1. A value of 1 indicates a perfect fit. CFI compares the fit of a null model (i.e., when unobserved variables are uncorrelated and independent) with the fit of the researcher's model [34]. A CFI value of greater than 0.90 shows a psychometrically acceptable fit to the data [30]. RMSEA is another quantitative value which describes how well the model fits the observed data. The value of RMSEA must be below 0.05 to show good fit.

## Results

### Principle component analysis

The Kaiser-Meyer-Olkin (KMO) analysis was carried out to examine the criteria of PCA for identifying the factor structure. Since KMO index was 0.89, the data set is suitable for factor analysis as it is greater than 0.50. Bartlett's test of sphericity was highly significant (χ^2 ^_(190) _= 2386; p = 0.00). This information allowed us to identify the factor model using the PCA approach. PCA of 20 items yielded a three factor model that accounted for 41.51% of the variance (Table 1).The first factor, which accounted for 22.17% of the variance had a factor weight ≥ 0.48. This factor, denoted by "compassionate care", is explained by ten items. The second factor, labelled "perspective taking", accounted for 9.93% of the variance. This factor includes four items. The final factor entitled "emotional detachment", accounted for 9.41% of the variance and consisted of three items. The remaining three items (1, 8 and 15) did not correlate with any one factor (did not appear to load on any of the factors), suggesting that these items may be inappropriate in their present form when used with medical students. Table 1 also shows the means and standard deviations for each item in the JSPE. We computed Pearson's correlation coefficients to investigate the inter-relationships between JSPE dimensions (factors). Correlations between each of the three factors (components) are outlined in Table 2. All of the correlation coefficients were significant and positively correlated with one another. Based on the magnitude of the coefficients, the strength of the association was greatest between perspective taking and compassionate care.

**Table 1:** Principle component analysis of items in the Jefferson Scale of Physician Empathy with communalities (h^2^) of each item (n = 853)*†

No.	Item	Factor 1	Factor 2	Factor 3	h^2^	Mean	SD
20	I believe that empathy is an important therapeutic factor in medical treatment.	0.65	0.00	0.00	0.46	6.03	1.03

14^‡^	I believe that emotion has no place in the treatment of medical illness.	0.64	0.00	0.00	0.43	6.05	1.14

16	Physicians' understanding of the emotional status of their patients, as well as that of their families is one important component of the physician-patient relationship.	0.60	0.00	0.00	0.43	5.90	1.00

12^‡^	Asking patients about what is happening in their personal lives is not helpful in understanding their physical complaints.	0.60	0.00	0.00	0.37	5.90	1.17

11^‡^	Patients' illnesses can be cured only by medical or surgical treatment; therefore, physicians' emotional ties with their patients do not have a significant influence in medical or surgical treatment.	0.59	0.00	0.00	0.36	5.60	1.39

7^‡^	Attention to patients' emotions is not important in history taking.	0.58	0.00	0.00	0.30	5.90	1.28

2	Patients feel better when their physicians understand their feelings.	0.55	0.00	0.00	0.34	6.50	1.24

19^‡^	I do not enjoy reading non-medical literature or the arts.	0.54	0.00	0.00	0.39	6.50	1.24

13	Physicians should try to understand what is going on in their patients' minds by paying attention to their non-verbal cues and body language.	0.51	0.00	0.00	0.32	5.88	1.03

10	Patients value a physician's understanding of their feelings which is therapeutic in its own right.	0.48	0.00	0.00	0.39	5.78	1.00

1^‡^	Physicians' understanding of their patients' feelings and the feelings of their patients' families does not influence medical or surgical treatment.	0.00	0.00	0.00	0.15	5.57	1.44

15	Empathy is a therapeutic skill without which the physician's success is limited.	0.00	0.00	0.00	0.21	5.50	1.37

8^‡^	Attentiveness to patients' personal experiences does not influence treatment outcomes.	0.00	0.00	0.00	0.21	5.61	1.27

17	Physicians should try to think like their patients in order to render better care.	0.00	0.69	0.00	0.44	4.75	1.49

9	Physicians should try to stand in their patients' shoes when providing care to them.	0.00	0.53	0.00	0.38	5.56	1.35

5	A physician's sense of humor contributes to a better clinical outcome.	0.00	0.51	0.00	0.30	4.90	1.33

4	Understanding body language is as important as verbal communication in physician patient relationships.	0.00	0.43	0.00	0.28	5.84	1.13

6^‡^	Because people are different, it is difficult to see things from patients' perspectives.	0.00	0.00	0.72	0.58	4.65	1.53

3^‡^	It is difficult for a physician to view things from patients' perspectives.	0.00	0.00	0.68	0.48	4.57	1.44

18^‡^	Physicians should not allow themselves to be influenced by strong personal bonds between their patients and their family members.	0.00	0.00	0.50	0.32	3.36	1.56

	% of variance	22.17	9.93	9.41			

	Alpha	0.79	0.44	0.37			

* The factor label components are as follows: F1, compassionate care; F2, perspective taking; F3, emotional detachment

^† ^The factor pattern coefficients of 0.40 and below were replaced by zeros

^‡ ^Items were reverse scored (strongly agree = 1, strongly disagree = 7)

**Table 2:** Correlation matrix of the Jefferson Scale of Physician Empathy dimensions (n = 853)

Dimension	Compassionate care	Perspective taking	Emotional detachment
Compassionate care		0.38^†^	0.09*

Perspective taking	0.39^†^		0.11*

Emotional detachment	0.09*	0.10^†^	

* p < 0.05

^† ^p < 0.01

### Confirmatory factor analysis

We performed CFA based on the variance-covariance matrix using the AMOS 17 statistical package for testing the three factor model [35]. Parameters were estimated for the CFA model based on the maximum likelihood procedure (sometimes called path analysis) involving fitting the variances and covariances among observed scores. AMOS therefore created a covariance matrix, including the variances and covariances among observed scores.

It was essential to identify the three factor model in order to estimate the model parameters. Factor loadings and the variances and covariances among the factors plus the variances and covariances among the errors were used to identify the three-factor model. In conducting CFA, no warning messages were received from AMOS regarding parameter estimates. Based upon this information, the three factor model passed the "rules" for identification [34].

**Figure 1:**
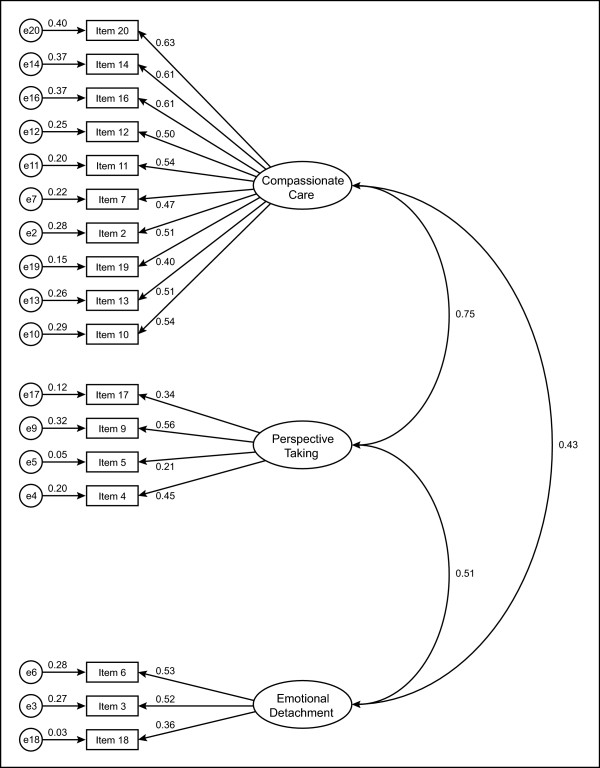
**Hypothesised 17-item model of factorial structure of the Jefferson Scale of Physician Empathy (n = 853)**.

Following identification of the three-factor model, we examined the assessment of universal fit pertaining to the quality of the model in AMOS in order to support or reject its appropriateness for the population examined. The next step was to illustrate the observed (items) and unobserved (factors) in the hypothesised model (Figure 1). The observed variables are represented as rectangles; ellipses represent the unobserved variables and the circles represent measurement error. The structural model consists of three interrelated constructs, including compassionate care, perspective taking and emotional detachment. The arrow between the unobserved variable and the observed variable represents a regression path and its number represents the standardised regression weight. The arrow between a small circle and the observed variable represents a measurement error term. The double-headed arrows represent the correlation between two unobserved variables (factor covariances) of the model.

Given that items 1, 8 and 15 had no significant factor loadings on any of the factors, they were excluded and a final model was proposed in which only the remaining 17 items were used to measure the underlying constructs of empathy. Thus, the 17-item JSPE model was separately tested for each data sample (total sample, female sample and male sample). Nonetheless, corrected item-total correlations were significant for these three items and they thus contribute to total scale score. In viewing the model shown in Figure 1, readers will note that empathy is represented as a multidimensional construct with compassionate care, perspective taking and emotional detachment, acting as conceptually independent factors. For example, the value 0.75 is the correlation between compassionate care and perspective taking. The values such as 0.63 and 0.61 are standardised regression weights.

In contrast to other the statistical hypothesis testing procedures, rejection of the null hypothesis in SEM does not support a research hypothesis. Focusing on Table 3, the significant χ^2 ^value (p = 0.00) does not imply support for the three-model factors in total sample and by gender. In other words, when a model has a good fit to the observed data, the p-value for chi-squared test is not significant. Therefore, one might conclude that the three-factor model in the total sample and by gender has no good fit. However, it should be noted that empirical studies showed that the p-value becomes significant if sample size is large enough [34,36,37]. Given that the interpretation of the chi-squared fit test is affected by large samples (as seen in this study), psychometric researchers employ the ratio of chi-square to degrees of freedom as a superior index for assessing a good model. A ratio of ≤ 2 indicates a superior goodness-of-fit between the three-factor model and the sample data [38].

**Table 3:** Goodness-of-fit indices for the three-factor model of the Jefferson Scale of Physician Empathy (n = 853)

Model	χ^2^	df	p	χ^2^/df	GFI	CFI	RMSEA
Total sample	228.04	116	0.00	1.77	0.97	0.95	0.03

Female	145.06	116	0.03	1.24	0.95	0.96	0.02

Male	178.06	116	0.00	1.53	0.96	0.94	0.03

Returning to Table 3, the χ^2^/df ratio is ≤ 2 in three data samples (total sample, female students and male students). This demonstrates that the three-factor model provides an adequate representation of the data, reflecting a good fit. Given the sensitivity of the chi-squared to sample size, a wide variety of other indices have been suggested to assess the adequacy of the model. In reviewing values of both CFI and GFI in Table 3, it is evident that the three-factor model represents a very good fit to the total sample and male and female students.

The RMSEA is another "fit" index that assesses the absolute fit of a model. A RMSEA of < 0.03 in three samples indicates good fit. It is apparent from the goodness-of-fit indices that the three-factor model provides the best fit to the observed data. A separate analysis was run to evaluate potential modifications for total sample, male students and female students. Based on small modification indices, all items were correlated.

### Reliability and known group validity

The Cronbach-alpha internal consistency estimate for the 20 items on the JSPE was 0.76. In comparison with other studies our coefficient alpha was lower than that reported for American and Japanese medical students (r_α _= 0.80) and higher than that reported in Mexican (r_α _= 0.74) students [1,18,39]. The values of alpha for male and female students was the same as the total sample (r_α _= 0.76), suggesting an acceptable reliability. Corrected item-total score correlations of the JSPE ranged from 0.11 to 0.54, and all were positive. Items 1, 8 and 15 that were not loaded on each component in the PCA had a corrected item-total correlation of 0.25, 0.35 and 0.34, respectively. These items also had a communality of 0.15, 0.21 and 0.21, respectively. This indicates that these items are unrelated to other items in the data set. In order to determine the degree to which the scale can demonstrate different scores for gender groups on each of the dimensions of the JSPE, known group validity was assessed.

The data from the independent samples t-test computed on each of the factors (dimensions) are presented in Table 4. Gender groups were significantly different on the 3 dimensions. Female students were more likely to display compassionate care, perspective taking and emotional detachment than male students (Table 4).

**Table 4:** Comparison between male (n = 351) and female (n = 470) responses on the Jefferson Scale of Physician Empathy*

Dimension	Male		Female		t
		
	Mean	SD	Mean	SD	
Compassionate care	5.70	0.70	6.08	0.58	7.12^‡^

Perspective taking	5.10	0.85	5.50	0.76	3.02^†^

Emotional detachment	4.08	0.99	4.40	0.98	2.20^†^

* 32 students did not indicate their gender

^† ^p < 0.05

^‡ ^p < 0.01

## Discussion

Given (a) the worldwide use of JSPE within the context of patient care and doctor-patient relationship and (b) the importance placed on the enhancement of level of empathy among medical students, it was considered important to validate the scale for use with this group. Previous research studies have examined the factor structure by EFA [1,18,39]. To our knowledge however, this is the first study assessing the three-factor model of the JSPE using SEM by specifying relationships among the observed variables and the unobserved variables.

Based on the data, we generated a three-factor model using Varimax rotation. To determine the psychometric properties of JSPE, the specification of the three-factor model (hypothetical factors); identification of the model; assessment of fit between the model and the observed variables (items) were presented through the CFA approach. Following specifying the three-factor model and model identification, fit statistics indicted that the model concurs with the data and provided the best fit with observed variables. The three-factor model has been well identified as each factor has at least three indicators (items) which is required in the model identification [40]. However, we have renamed the "ability to walk in the patient's shoes" factor as "emotional detachment" since we feel that the items that have loaded into this third factor in our study are best explained by this concept.

Further model assessment shows that the hypothesised three-factor model of JSPE structure is well fitting across the gender differences of medical students. Consequently, we could conclude that the 20 item JSPE provides a valid and reliable scale to measure medical students' attitudes toward empathy. The results also supported that the scale is valid and reliable to measure the level of empathy in both male and female students. Therefore, medical educators can use the JSPE in order to assess medical students' attitudes regarding empathy. The results from such assessments can be used to modify medical education programmes aiming at enhancing empathy.

This study has been unable to compare the findings of the study with previous research studies as there are no studies undertaking the CFA approach and SEM to validate the JSPE among medical students. For this reason, further studies which compare the findings of this study and test the fitness of the three-factor model will need to be undertaken. Given the present study supported a multidimensional conceptualisation with eigenvalue > 1.25, it does not impede researchers from adopting other models including the unidimensional model. The findings of this study encourage medical education researchers to consider this scale for empathy enhancement among not only undergraduate and graduate medical education programmes but also practising doctors.

### Limitations of the study

The findings of this study were based on data gathered from a single institution, arguably a sample of convenience. The findings may be somewhat limited in generalisability owing to their derivation from only a single medical school. The entirety of the sample is nonetheless representative of students from diverse multicultural and social backgrounds which may mitigate the aforementioned limitation. In addition, the PCA generated a hypothesised factor structure for the data set, which is confirmed on the same data set by the CFA. Because the same data set is used to both generate and then confirm the factor structure it may be less informative. Therefore CFA should be conducted with a different data set using the hypothesised factor structure. We wished to perform a secondary CFA in another school but it was not practically feasible. We hope that other schools in the UK use CFA in order to test the model by using their own data set.

Finally, one cannot overemphasise the limitations of self-reported data as this may limit the validity of findings. Respondents for various reasons may under or overestimate the practice of empathy. A methodological problem frequently associated with the use of self-report measures, which may have been evident in the present study, is the inability to determine the extent to which responses accurately reflects the respondents' experiences and expectations of their empathy due to social desirability and inaccurate recall.

## Conclusions

The results of this study supported the usefulness of the JSPE as a brief, reliable and psychometrically sound scale for measuring empathy among medical students in relation to their patients. Moreover, a valuable model of the dimensional structure of empathy emerged, highlighting compassionate care, perspective taking and emotional detachment. This model can provide directions to enhance empathy in the context of medical education. We recommend that other medical schools, not just in the UK, to use the SEM in order to test the model by using their own data set. The results of this study further illustrate the utility of this method in the analysis of empathy item data.

## Competing interests

The authors declare that they have no competing interests.

## Authors' contributions

All authors contributed equally to the conception and design of the study as well as the acquisition, analysis and interpretation of data and final drafting of the manuscript. All authors contributed to the critical revision of the paper and approved the final paper for publication.

## Pre-publication history

The pre-publication history for this paper can be accessed here:

http://www.biomedcentral.com/1472-6920/11/54/prepub
